# Charge Storage by Electrochemical Reaction of Water Bilayers Absorbed on MoS_2_ Monolayers

**DOI:** 10.1038/s41598-019-40672-w

**Published:** 2019-03-08

**Authors:** Ruihua Zhou, Sufeng Wei, Yan Liu, Nan Gao, Guoyong Wang, Jianshe Lian, Qing Jiang

**Affiliations:** 1 Key Laboratory of Automobile Materials, Department of Materials Science and Engineering, Jilin University, Changchun, 130025 PR China; 2 Key Laboratory of Advanced Structural Materials, Changchun University of Technology, Changchun, 130012 PR China; 3 Key Laboratory of Bionic Engineering (Ministry of Education) and State Key Laboratory of Automotive Simulation and Control, Jilin University, Changchun, 130022 PR China; 4 College of Mechanical and Vehicular Engineering, Changchun University, Changchun, PR China

## Abstract

It is well-known that in neutral and acidic aqueous electrolytes, MoS_2_ monolayers can store charges by adsorption of cations on to the electrode-electrolyte interface as its analog of graphene. Restricted by its low conductivity and the charge storage mechanism, the electrochemical performance of MoS_2_ monolayer supercapacitor electrode is not satisfactory. It is reported here that water bilayers absorbed on MoS_2_ monolayers can be involved in charge storage. One proton of each absorbed water molecule can intercalate/de-intercalate the water bilayers during charging/discharging in the alkaline aqueous electrolyte. For two water molecules are present for every Mo atom, the water bilayers can endow MoS_2_ monolayers an ultrahigh specific capacitance. In this paper, 1T phase MoS_2_ nanosheets with three monolayers were synthesized by hydrothermal reaction. It presents a specific capacitance of 1120 F g^−1^ at a current density of 0.5 A g^−1^ in KOH. As it is assembled with active carbon into a hybrid supercapacitor, the device has an energy density of 31.64 Wh kg^−1^ at a power density of 425 W kg^−1^, and gets a specific capacitance retention of 95.4% after 10,000 cycles at 2 A g^−1^.

## Introduction

**Figure 1:**
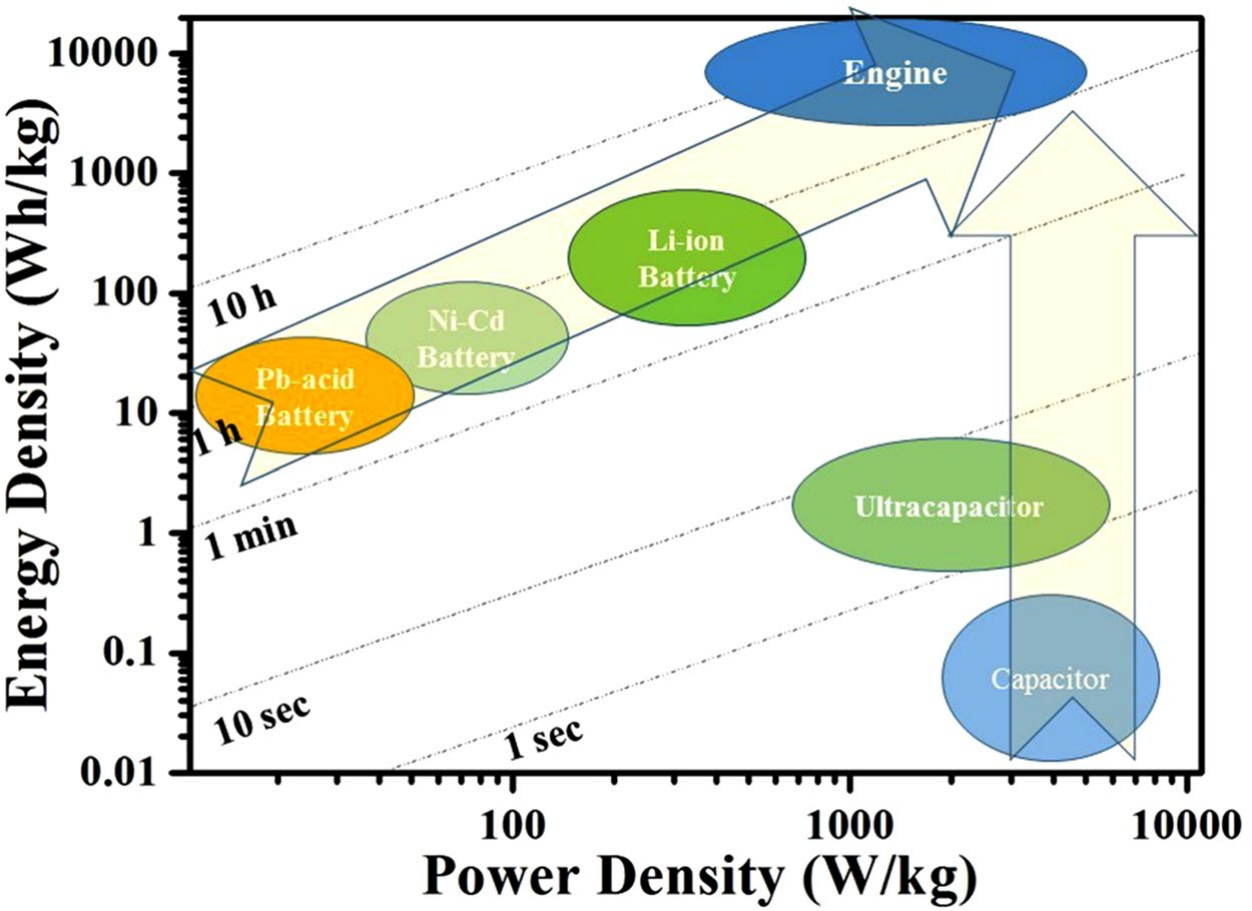
Ragone plot for electrochemical energy storage devices and traditional internal-combustion engine. Times shown are the time constants of the devices, obtained by dividing the energy density by the power density.

The development of renewable energy storage devices is one of the most promising ways to address the current energy crisis along with the global environmental concern and pushes scientific communities to search for sustainable energy storage technologies^1,2^. Among the various realistic solutions, energy can in particular be stored electrochemically in batteries and supercapacitors. Batteries present a high energy density, which can keep our devices working throughout the day^3^. Supercapacitors, owning to their high power density and long cycle life, have wild applications in regenerative braking and loading leveling system of cars and electric mass transit vehicles that would otherwise lose their braking energy as heat^4^. During the past several years, many researchers have engaged to find ideal electrode materials which might combine the high energy density of batteries and the short charging time of supercapacitors, as shown in Fig. 1^5,6^. Obviously, electric vehicles (EV) equipped with such devices would have short charging time, excellent acceleration performance and grade ability, and long cruising mileage, which may be as efficient and convenient as traditional internal-combustion engine vehicles but more eco-friendly. While some researchers tend to approach these goals by increasing the power density of batteries (for instance, many nanostructured lithium-ion battery electrode materials show improved rate performance for the diffusion paths in the solid electrode materials are shortened^7^), the other researchers tend to approach these goals by increasing the energy density of supercapacitors (lots of nanoscaled Ni(OH)_2_ analogous materials which are generally cathode materials of Ni-MH batteries have been applied in C//Ni(OH)_2_-based hybrid supercapacitors and achieved very high energy density comparable to lithium-ion batteries^8,9^). Supercapacitors, also known as electrochemical double layer capacitors (EDLCs), store charge by adsorption of electrolyte ions on to the electrode–electrolyte interface^6^. In order to achieve full utilization of electrode materials, many methods have been applied on carbon materials (enhancing the specific surface area, optimizing the porous structure and morphology^10,11^) to enhance the proportion of the accessible surface atoms in electrode materials. Thus, single wall carbon nanotubes and graphene, of which all atoms are on the surface, have been considered as promising electrodes for supercapacitors, and capacitances around 100 to 200 F g^−1^ have been achieved on such materials^12–15^. Besides the accessible surface atoms, near-surface atoms of pseudocapacitance materials are also involved in energy storage process. They display much higher specific capacitance than EDLCs for more proportion atoms in such materials contribute to charge storage. A new type of charge storage mechanism for supercapacitors, intercalation pseudocapacitance, was reported recently^16–18^. Cations intercalate/de-intercalate the bulk of the active materials, but doesn’t cause phase transition and is not limited by the bulk diffusion of cations^18^. Compared to EDLCs and pseudocapacitance, bulk atoms, besides surface atoms and/or near-surface atoms, in electrode materials can be used to store charge. Thus the highest utilization of electrode materials endows intercalative pseudocapacitance with the highest specific capacitance. Generally speaking, the utilization and efficiency of each atom in electrode materials determine the unit of capacity (mAh g^−1^). Theoretically, each atom of battery electrode materials can be used, although in a low rate mode, so that they present the highest unite of capacity. That is to say, the ideal electrode materials which can fast charge/discharge a huge amount of charges as shown in Fig. 1 may also combine the charge storage mechanisms of both battery and capacitor. It needs battery reaction (intercalation/de-intercalation of ions into/from the matrix) to enhance the capacity, meanwhile it also needs the capacitor reaction close to surface in order to enhance charging/discharging rate.

Two-dimensional transition–metal dichalcogenides have emerged as a fascinating new class of materials for wild applications^19–21^. Among them, the ultrathin MoS_2_ nanosheets (a new inorganic graphene analog) have recently evoked enormous research enthusiasm as electronics/optoelectronics^22,23^, sensors^24^, energy-storage and conversion devices^25–27^. However, the very low conductivity of the trigonal prismatic (labeled as 2H) phase MoS_2_ monolayers, which is thermodynamically stable, put sands in the wheels of their application as supercapacitors^19^. 2H-MoS_2_ monolayers can also store charges by interfacial adsorption as the analog of graphene. But the capacitance is much inferior to graphene^28–30^ for the conductivity (10^–6^–10^–5^ S cm^−1^) is much lower than that of graphene (~100 S cm^−1^)^26^. In order to improve the electrochemical performance, the 2H-MoS_2_ monolayers was even composited with graphene or CNTs to ameliorate the conductivity, and with pseudocapacitive materials such as polypyrrole to enhance the capacitance^31–34^. The octahedral (labeled as 1T) phase MoS_2_ monolayers, which is a thermodynamically metastable isomer of 2H phase, is 10^7^ times more conductive than the semiconducting 2H phase^19,35,36^. Thus, the capacitor electrode composed of 1T-MoS_2_ monolayers presented impressive electrochemical performance and very high capacitance^26^. It has been reported and evidenced by X-ray diffraction for decades that as MoS_2_ monolayers are in aqueous solution, a new phase is obtained where two water monolayers are present and sandwich the MoS_2_ monolayers^37,38^. However, according to the recent reports on MoS_2_ monolayer supercapacitors using neutral and acidic aqueous electrolytes, they don’t contribute to, or even affect, the charge storage mechanism and performance^26^. It is reported in this paper that as tested in alkaline aqueous solution, the water bilayers can endow the MoS_2_ nanosheets, both metallic 1T phase and semiconducting 2H phase, a superior electrochemical energy storage performance. The charge storage mode also changes from low-energy EDLC mode to high-energy battery-type mode. The electrode materials composed of self-assembled rose-like metallic 1T phase MoS_2_ nanosheets present a very high specific capacitance (1120 F g^−1^) at 0.5 A g^−1^. This is the highest value for pure MoS_2_ as far as we know. The MoS_2_ nanosheets as a cathode were assembled with active carbon to form a MoS_2_//AC hybrid supercapacitor. Owning to the high capacitance of the 1T-MoS_2_ and the wide voltage window, the hybrid capacitor shows an impressive energy density of 31.64 Wh kg^−1^ in KOH aqueous electrolyte, which is even comparable to traditional lithium-ion batteries. The phenomenon reported in this paper is sure to expand the application of MoS_2_ nanosheets in electrochemical energy storage.

## Results and Discussion

### Characterizations

**Figure 2:**
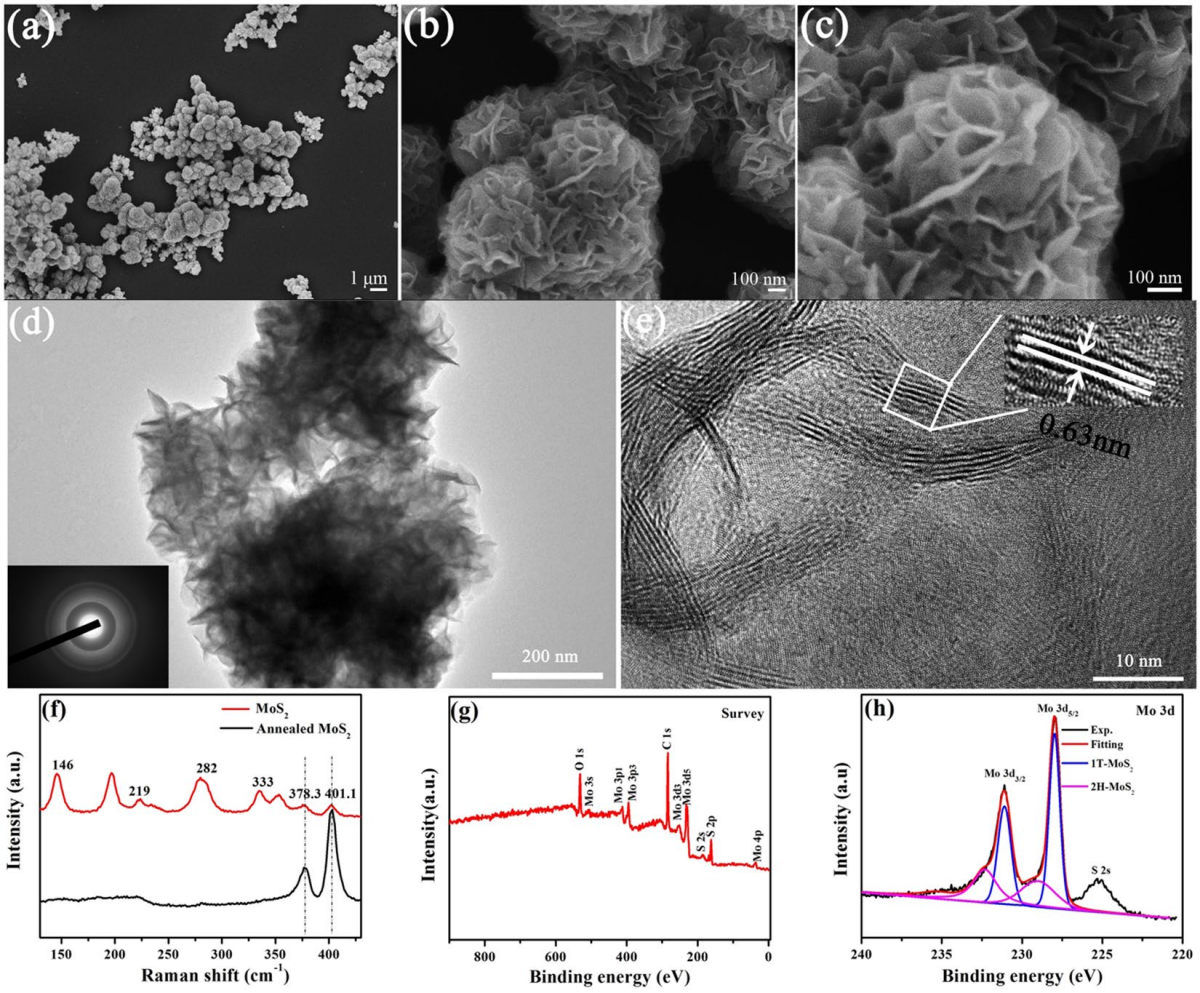
The morphology of the as-synthesized MoS_2_ particles observed by FESEM at low (**a**), middle (**b**), and high (**c**) magnifications. (**d**) A TEM image of the as-synthesized MoS_2_ particles. The inset is the corresponding SAED pattern. (**e**) A HRTEM image of the as-synthesized MoS_2_ nanosheets. The interference fringes of (002) plane indicate each nanosheet contains 2–4 MoS_2_ monolayers. (**f**) Raman spectra of the as-synthesized MoS_2_ nanosheets and the annealed MoS_2_ nanosheets. (**g**) An X-ray photoelectron survey spectrum of the as-synthesized MoS_2_ nanosheets. (**h**) A high-resolution X-ray photoelectron spectrum of Mo 3d region. Contributions from 1T and 2H phase components in the Mo 3d spectrum are indicated by blue and magenta curves, respectively.

The morphology of the as-prepared MoS_2_ nanosheets was carefully observed by FESEM and TEM, and the corresponding images are shown in Fig. 2. The nanosheets are wrinkled, and have a diameter of 100 nm and a thickness of 6 nm, as shown in Fig. 2b,c. The wrinkled sheets are self-assembled into rose-like spherical particles with a diameter of about 800 nm, leaving millions of nanopores between them. The interference fringes of the nanosheets were clearly shown in the HRTEM image of Fig. 2e. The spacing between each fringe is 0.63 nm, corresponding to the spacing of MoS_2_ (002) crystal plane. For the spacing of (002) crystal plane is half of (001) crystal plane and each sheet contains 4–8 interference fringes of (002) crystal plane, each sheet has 2 to 4 MoS_2_ monolayers. The ultrathin structure of the nanosheets results in a low crystallinity indicated by the Debye-Scherrer ring in the inset of Fig. 2d. However, the ultrathin and wrinkled nanosheets endow the rose-like nanoparticles a high BET specific surface area (54.7 m^2^ g^−1^), as shown in Fig. S4. According to the BJH pore-size distribution results in the inset of Fig. S4, the nanopores between the nanosheets in the rose-like particles have a very wide size distribution from 20 to 80 nm.

The phase structure of the MoS_2_ nanosheets was further investigated by Raman spectroscopy, because Raman spectrum is very sensitive to the symmetry of the sulfur in the matrix and effective to differentiate 1T-MoS_2_ and 2H-MoS_2_^39,40^. As shown in Fig. 2f, besides the depressed typical Raman shifts at 378.3 and 401.1 cm^−1^ for ${{\rm{E}}}_{2{\rm{g}}}^{1}$ and A_1g_ of MoS_2_, obvious Raman shifts appear at 146, 219 and 333 cm^−1^ in the spectrum of the as-prepared sample, which is associated with the phonon modes in 1T-MoS_2_^41,42^. It implies that the as-prepared nanosheets contain lots of 1T phase. However, only ${{\rm{E}}}_{2{\rm{g}}}^{1}$ and A_1g_ appear in the spectrum of the annealed MoS_2_, indicating the total transition of the 1T-MoS_2_ to 2H-MoS_2_ after annealing^26^. The number of the monolayers in MoS_2_ film can be calculated by the Raman frequency difference between A_1g_ and ${{\rm{E}}}_{2{\rm{g}}}^{1}$ modes^43^. It is about 22.8 cm^−1^, indicating that the sheet has three monolayers according the ref.^43^. This result is consistent with the HRTEM observation. The 1T and 2H phase compositions in the MoS_2_ nanosheets were further identified by XPS. Two predominant peaks Mo and S appear in the survey spectrum of Fig. 2g. The S to Mo atomic ratio of the as-prepared MoS_2_ nanosheets is ~2.1 according to the XPS element detection. It has been reported that 1T phase can cause ~1 eV chemical shifts to lower binding energy on both Mo 3d peaks and S 2p peaks^44^. So the high-resolution XPS of Mo 3d peaks and S 2p peaks were investigated and the corresponding spectra are shown in Fig. 2h and S5, respectively. As shown in the high-resolution XPS spectrum of Mo 3d peaks in Fig. 2h, the Mo 3d spectra consist of two peaks located at 228.0 and 231.1 eV corresponding to Mo^4+^ 3d_5/2_ and 3d_3/2_ components of 1T-MoS_2_, respectively^26^. Both peaks are accompanied by weak shoulders at higher binding energies of 229.0 and 232.1 eV, which are the typical sites for Mo^4+^ 3d_5/2_ and Mo^4+^ 3d_3/2_ of 2H-MoS_2_. The high-resolution XPS spectrum of S 2p peaks in Fig. S5 also implies a plenty of 1T-MoS_2_ in the rose-like particles. The relative content of 1T phase components in the as-prepared MoS_2_ nanosheets was calculated to be about ~78.0% according to the relative peak height of Mo 3d peaks in Fig. 2h.

### Electrochemical analysis

**Figure 3:**
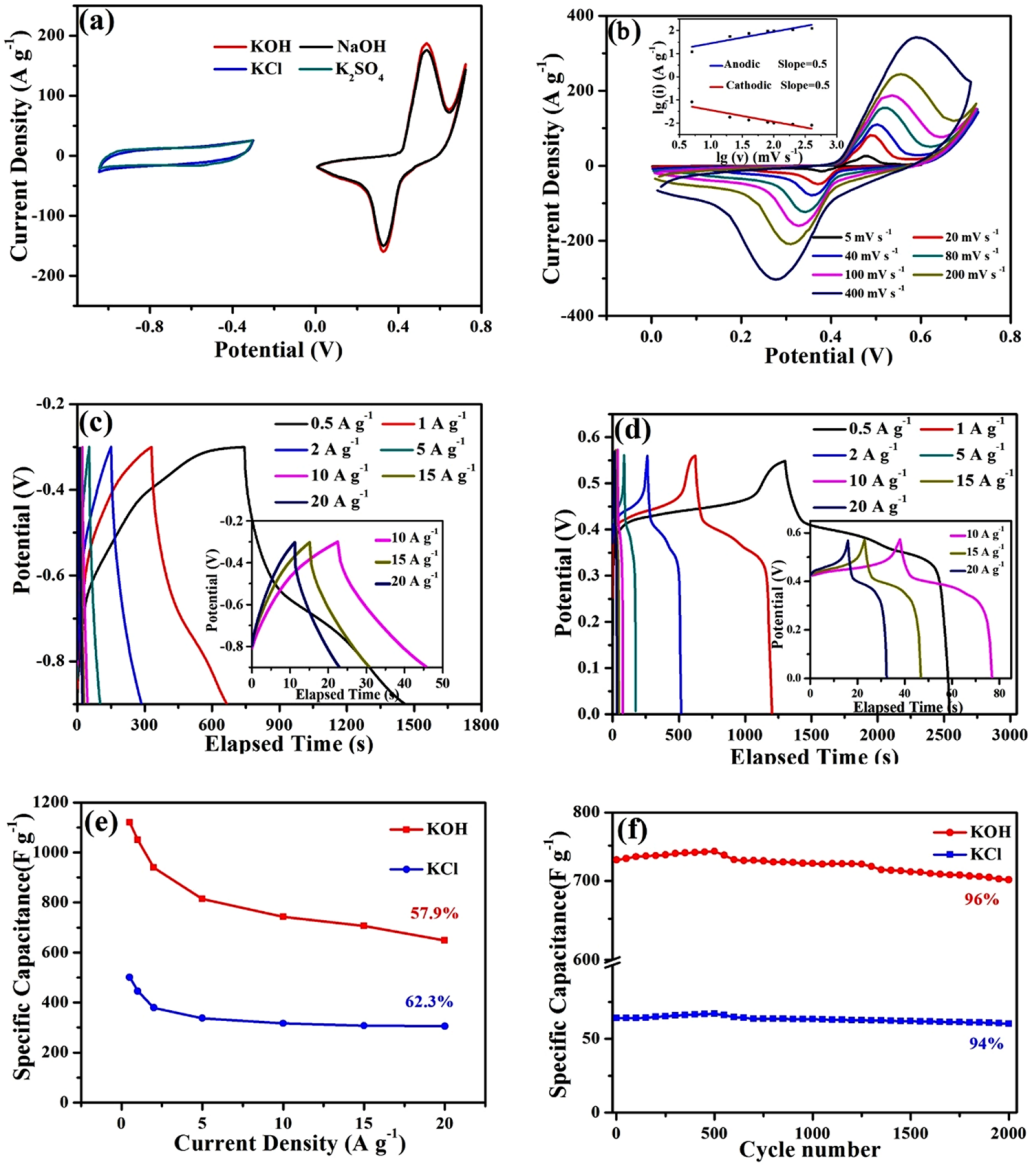
(**a**) CV curves of the 1T-MoS_2_ electrodes at a scan rate of 100 mV s^−1^ in 3 M KOH, 3 M NaOH, 3 M KCl and 0.5 M K_2_SO_4_, respectively. (**b**) CV curves of the 1T-MoS_2_ electrodes at various scan rates from 0 to 0.75 V in 3 M KOH. The inset is the logarithmic relationship between the current densities (i) at a fixed potential and the scan rate (v) in the CV curves. (**c**) The GCD curves of the 1T-MoS_2_ electrodes at various current densities from 0.5 to 20 A g^−1^ in 3 M KCl; (**d**) The GCD curves of the 1T-MoS_2_ electrodes at various current densities from 0.5 to 20 A g^−1^ in 3 M KOH. (**e**) The specific capacitance evolutions of the 1T-MoS_2_ electrodes with current density in 3 M KOH and 3 M KCl, respectively. (**f**) The cycling performances of the 1T-MoS_2_ electrodes at a current density of 10 A g^−1^ in 3 M KOH and 3 M KCl, respectively.

The capacitive behavior of the 1T-MoS_2_ electrode was firstly investigated in 3 M KCl electrolyte in a potential window of −1.05 to −0.3 V using CV measurement in a three-electrode configuration where SCE and Pt plate served as the reference and counter electrodes, respectively. As shown in Fig. 3a, the resulting CV curve in KCl presents a horizontal straight line without any redox peaks. We substituted Cl^−^ with SO_4_^2−^ in electrolyte and further investigated the capacitive behavior in 0.5 M K_2_SO_4_ aqueous solution. The CV curve remains the same and is overlapped with the one in KCl. It implies that anion substitute in electrolyte has no effect on the capacitive behavior of the 1T-MoS_2_ electrode. The capacitive behavior of the 1T-MoS_2_ electrode is totally determined by cation adsorption (here is K^+^ in both electrolytes) on the electrode-electrolyte interface. However, as the anion is further substituted by OH^−^ and the CV method is carried out in 3 M KOH, the resulting CV curve is changed to battery-type: a typical anodic peak occurred at 0.53 V and a typical cathodic reduction peak occurred at 0.33 V are detected in the potential window of 0 to 0.75 V, respectively. The area encircled by the CV curve is dramatically enlarged compared to that in K_2_SO_4_ and KCl, which indicates a higher specific capacitance. A straight substitution of K^+^ for Na^+^ doesn’t cause any further change on the CV curve. As shown in Fig. 3a, the CV curve tested in KOH overlaps the one tested in NaOH. It implies that the capacitive behavior tested in alkaline aqueous electrolyte is determined by OH^−^ and has no relationship with the kind of cation. The CV curves at various scan rates in KOH (Fig. 3b) also indicate that a battery-type charge storage behavior happens in the 1T-MoS_2_ electrode. The potential difference between the anodic peaks and the cathodic peaks on the CV curves becomes larger and larger with scan rates. What’s more, it is very obvious in the inset of Fig. 3b that the relationship between the current density (i) at a fixed potential and the scan rate (v) is presented by i = av^b^, and b is close to 0.5^45^. The GCD measurements also support this point of view. As the electrodes are tested in KCl, the obtained curves show quasi-triangular shapes, which reflect the charge accumulation process with voltage as an EDLC (Fig. 3c). As the electrodes are tested in KOH, the obtained curves always show a plateau at a certain voltage, which is a general phenomenon in batteries and reflects the phase-transformation reactions in batteries (Fig. 3d). The transition of charge storage mechanism in alkaline electrolyte is not the unique phenomenon belonging to 1T-MoS_2_ nanosheets. After 4 hours annealing at 800 °C, 1T-MoS_2_ nanosheets can transform into 2H-MoS_2_ nanosheets^26^, as proved by the Raman spectrum in Fig. 2f ^41^. The transition of charge storage mechanism is also found on 2H-MoS_2_ electrode, as shown in Fig. S7. As the scan rate increases up to 400 mV s^−1^, the shape of the CV curves tested in KCl (Fig. S6b) and K_2_SO_4_ (Fig. S6c) retain their quasi-rectangular shape, but the potential difference between the anodic peaks and the cathodic peaks on the CV curves tested in KOH (Fig. 3b) and NaOH (Fig. S6a) become larger and larger. The specific capacitance was calculated based on the CV measurement and the trends of the specific capacitance with scan rate are summaries in Fig. S6d. It is very clear in this plot that the curves tested in OH^−^ electrolytes (KOH and NaOH) overlap with each other very well and the curves tested in neutral K^+^ electrolytes (KCl and K_2_SO_4_) also overlap with each other very well. However, the curves tested OH^−^ electrolytes are dramatically larger than that in neutral K^+^ electrolytes. And similar phenomenon is also found in 2H-MoS_2_ electrode (Fig. S7f). Those phenomena further testify OH^−^ can take change on charge storage ability and behavior of MoS_2_ in aqueous electrolyte. The plots in Fig. S6d imply that OH^−^ electrolytes can endow the MoS_2_ a much higher specific capacitance in a wide range of scan rates, although it will descend more dramatically than that in neutral K^+^ electrolytes.

**Figure 4:**
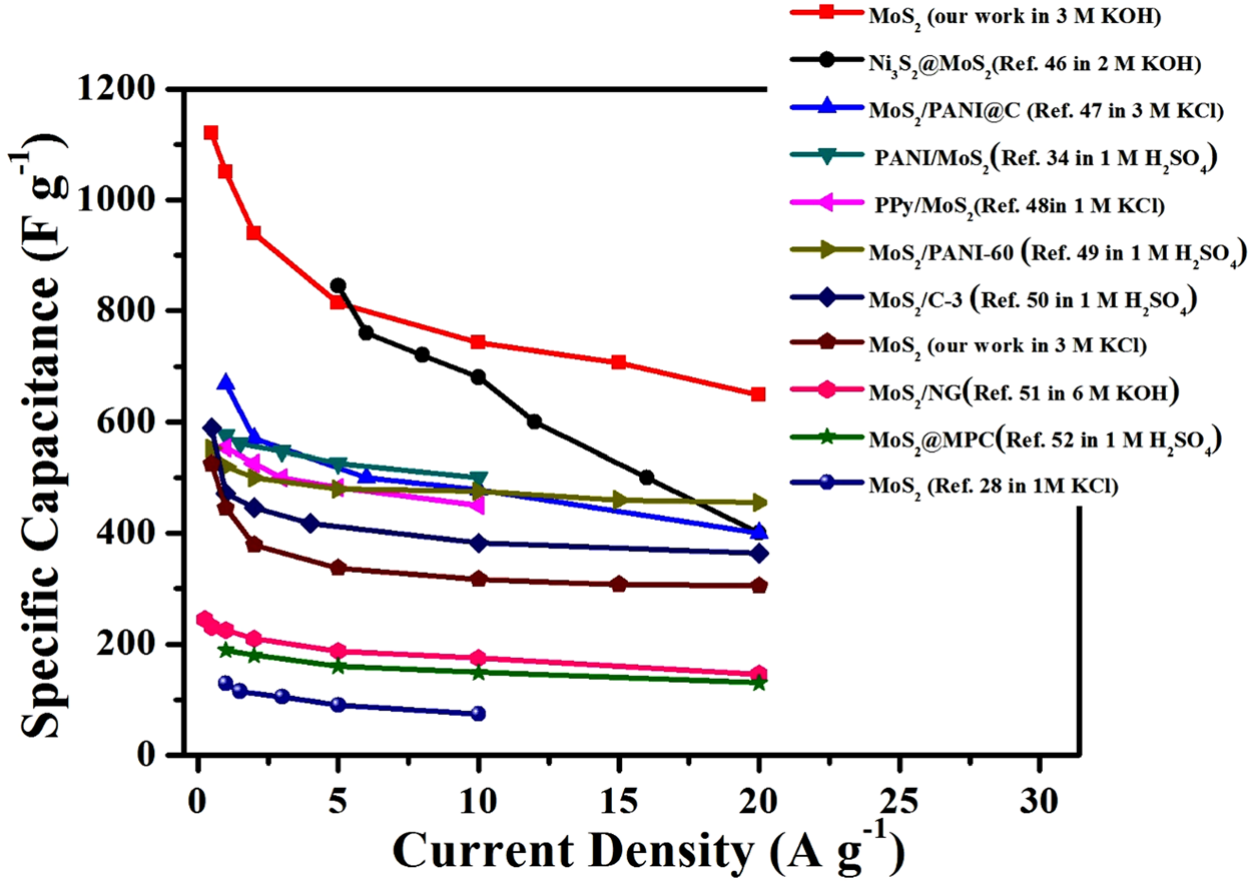
Capacitance comparison of MoS_2_-based electrode materials including our work and other reported works.

The capacitance of the 1T-MoS_2_ electrode was further verified by GCD measurement in KCl (Fig. 3c) and KOH (Fig. 3d) electrolytes within a current density ranging from 0.5 to 20 A g^−1^, respectively. The discharging time of the electrodes in both electrolytes decline as the current density increases from 0.5 to 20 A g^−1^. But at a certain current density, the discharging time of the electrode in KOH is always longer than that in KCl. The specific capacitance of the MoS_2_ electrode in both KOH and KCl electrolytes at different current density was calculated based on the GCD measurements, and the results are summarized in Fig. 3e. Benefiting from the good conductivity of 1T phase, the MoS_2_ electrode in KCl presents a very high specific capacitance of 483 F g^−1^ at 0.5 A g^−1^. It is higher than most of the pure MoS_2_ nanosheets electrodes, as shown in Fig. 4 ^28,34,46–52^, including the 1T phase MoS_2_ monolayer electrode which is 130 F g^−1^ in H_2_SO_4_^26^. But the specific capacitance in KOH is even higher. It reaches as high as 1120 F g^−1^ at 0.5 A g^−1^, which is the highest value for pure MoS_2_ so far. As the current density increases 40 times to 20 A g^−1^, 57.9% of the specific capacitance in KOH (648 F g^−1^) is retained, compared to 62.3% in KCl (300 F g^−1^). Although the specific capacitance in KOH declines more dramatically than that in KCl, it is always higher than that in KCl at each current density. In the end, it is still more than twice as much as that in KCl. In fact, the specific capacitance in KOH is higher than any reported specific capacitance of MoS_2_ related electrodes, even as MoS_2_ was composited with some pseudocapacitive materials with high specific capacitance or with some EDLC materials with high conductivity such as graphene, CNTs and so on, as shown in Fig. 4 ^28,34,46–52^. The cyclability of the 1T-MoS_2_ electrodes in both in KOH and KCl electrolytes was tested at a current density of 10 A g^−1^ over 2,000 cycles. The electrodes retained a capacitance of 96% in KOH and of 94% in KCl after 2,000 cycles, as shown in Fig. 3f. The charge transfer characteristics of the MoS_2_ electrodes in both KCl and KOH electrolytes were studied by EIS within the frequency range of 10^6^ and 10^−2^ Hz. And the corresponding Nyquist plots are shown in Fig. S8. The intersecting point of a small semicircle with real axis represents internal resistance (Rs) and its diameter represents interfacial charge transfer resistance (Rct)^53,54^. The Rs of MoS_2_ electrodes in both electrolytes are very close, about 0.3 Ω in KOH and 0.4 Ω in KCl. But the Rct in KCl (0.6 Ω) is twice the one in KOH (0.3 Ω).

**Figure 5:**
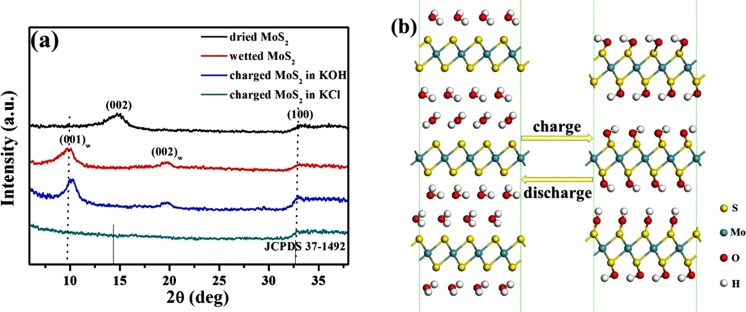
(**a**) XRD patterns of the 1T-MoS_2_ nanosheets at dried, wetted, charged in KOH and charged in KCl states. (**b**) Schematic illustration of the charge storage mechanism of the water bilayers absorbed on MoS_2_ monolayers during charging/discharging process.

In order to uncover the charge storage mechanism, the crystallographic structure of the 1T-MoS_2_ nanosheets at four different states (dried, wetted, wetted after charging in KOH and wetted after charging in KCl) were characterized by XRD, and the corresponding XRD spectra are shown in Fig. 5a. The dried MoS_2_ nanosheets present a typical powder diffraction pattern which matches very well with the standard powder diffraction file JCPDS card #37-1492. After it is wetted by aqueous solution, two dramatic diffraction peaks at 2θ of 9.77° and 19.17° appeared instead of (002) peak of MoS_2_ in the spectrum. These two peaks have been reported for decades. They belong to the water bilayers sandwiching MoS_2_ layers^37^, as denoted by the labels in the spectrum. The number of water molecules in the water bilayers was carefully investigated by weighing measurment in the ref.^37^, and it is found that two water molecules are present for every Mo atom. After charging in KOH, both water peaks slightly shift to high 2θ, at 2θ of 10.25° and 19.65°, respectively. This shift indicates constriction of water bilayers. The spacing of water bilayer (001) plane decreases by 0.43 Å from 9.05 Å to 8.62 Å. However, the water peaks are totally disappeared in the spectrum of wetted MoS_2_ nanosheets charged in KCl. Based on the electrochemical measurements and XRD characterization, we speculate that in the alkaline aqueous electrolyte, the water bilayers in wetted MoS_2_ nanosheets are involved in the charge storage process following the below equation:

1 $${{\rm{MoS}}}_{2}\cdot 2{{\rm{H}}}_{2}{\rm{O}}+2{{\rm{OH}}}^{-}\leftrightarrow {{\rm{MoS}}}_{2}\cdot 2{\rm{OH}}+2{{\rm{H}}}_{2}{\rm{O}}+2{{\rm{e}}}^{-}$$

And the corresponding charge storage process is schematically illustrated by Fig. 5b. A water monolayer should be absorbed on each side of MoS_2_ monolayer as it is wetted by an aqueous solution. And each S atom in the MoS_2_ monolayer should absorb one H_2_O molecule. During charging, a proton would be pulled away from each H_2_O molecule, which of course leads to the constriction of water bilayers. The proton could also return during the discharging process, as shown in Fig. 5b. That is to say, MoS_2_ can store charge analogous to aqueous Ni-MH battery electrode materials such as Ni(OH)_2_ and Co(OH)_2_^55^. The charge storage of MoS_2_ in alkaline aqueous solution mainly depends on the intercalation/de-intercalation of proton within the water bilayers. However, the MoS_2_ monolayer may be the fastest battery for proton needn’t diffuse within the crystalline framework during charging/discharging process. We also calculated the theoretical specific capacitance according to equation (1) assuming a potential window of 0.75 V, which is 1607 F g^−1^. The measured value of GCD measurement in KOH at 0.5 A g^−1^ is very close to, but still below, the theoretical value. The absorption may be very weak. The vanishment of water peaks in the XRD pattern after charging in KCl, as shown in Fig. 5a, implies that cation absorption on the MoS_2_ monolayer can effectively destroy the water bilayers.

### Electrochemical performance of the MoS_2_//AC hybrid supercapacitor device

**Figure 6:**
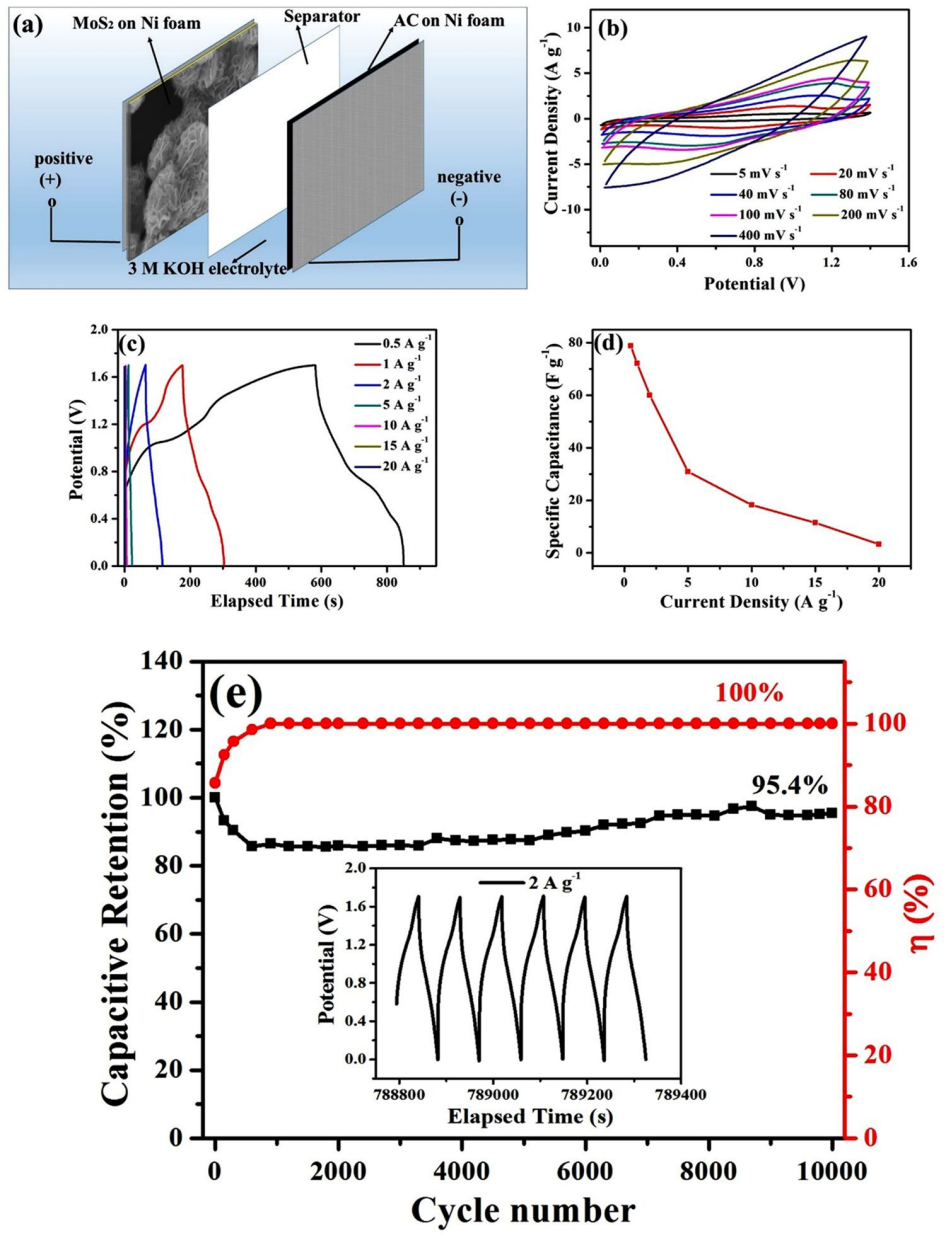
(**a**) Schematic illustration of the configuration of the MoS_2_//AC hybrid supercapacitor. (**b**) CV curves of the hybrid supercapacitor at various scan rates from 0 to 1.4 V. (**c**) GCD curves of the hybrid supercapacitor at various current densities from 0 to 1.7 V. (**d**) The specific capacitance evolution with current density. (**e**) Cycling performances and coulombic efficiency of the hybrid supercapacitor at a current density of 2 A g^−1^ in 3 M KOH electrolyte. And the inset is a section of GCD curves close to the end.

To further evaluate the MoS_2_ electrode for practical applications, a hybrid supercapacitor device was fabricated using the MoS_2_ as positive electrode and AC as negative electrode in 3M KOH aqueous electrolyte (denoted as MoS_2_//AC), as schematically illustrated by Fig. 6a. The CV curves of the device in Fig. 6b exhibit close EDLC properties, indicating a capacitor behavior. And the CV curves didn’t show obvious distortion at the scan rate up to 400 mV s^−1^, demonstrating a good rate character. The GCD curves of the device at a current density ranging from 0.5 to 20 A g^−1^ in a potential window of 1.7 V in Fig. 6c also present analogous triangular shape. The specific capacitance based on GCD measurements is summarized in Fig. 6d. The specific capacitance presents a very high value of 78.82 F g^−1^ at a current density of 0.5 A g^−1^, corresponding to an energy density of 31.64 Wh kg^−1^ at a power density of 425 W kg^−1^, which is comparable to the Nickel hydroxide related composite electrodes in reports (Table S1)^8,56–75^. The GCD measurements at 2 A g^−1^ were also performed on this device up to 10,000 cycles, and the corresponding evolution of the capacitance and coulombic efficiency is shown in Fig. 6e. And a section of the GCD curve close to the end is also shown in the inset. After initial hundreds of cycles, both the capacitance and coulombic efficiency enters into a relatively stable stage. After 10,000 cycles, the GCD curves still remain their quasi-triangular shape, and the device still possesses a great specific capacitance retention of 95.4%.

## Conclusion

In conclusion, three-layer 1T phase MoS_2_ nanosheets were fabricated via a one-pot facial hydrothermal reaction in this paper. These nanosheets were self-assembled into rose-like nanoparticles. It is demonstrated that in alkaline aqueous electrolyte the water bilayers absorbed on MoS_2_ layers can contribute to energy storage by proton intercalation/de-intercalation. During charging, a proton will break away from every water molecule in the water bilayers. During discharging, the proton will come back to form water molecules and water bilayers again. The proton intercalation/de-intercalation process endows MoS_2_ a very high capacity. The theoretical unit of capacity for MoS_2_ was calculated to be 334 mAh g^−1^. For a potential window of 0.75 V, it is corresponding to a capacitance of 1607 F g^−1^. Thus, in alkaline electrolyte, the as-prepared 1T phase MoS_2_ nanosheets can present an ultrahigh special capacitance of 1120 F g^−1^ at a discharge current density of 0.5 A g^−1^. As the pure 1T phase MoS_2_ nanosheets were assembled into MoS_2_//AC hybrid supercapacitor device, it presented a high energy density of 31.64 Wh kg^−1^ at a power density of 425 W kg^−1^, which is comparable to the Nickel hydroxide related composite electrodes in reports. The research in this paper is sure to expand the application of MoS_2_ nanosheets in electrochemical energy storage.

## Experimental Section

### Chemicals

Molybdenum (VI) oxide powder (MoO_3_), thioacetamide, urea and sodium chloride (NaCl) were purchased from Sinopharm Chemical Reagent Co., Ltd. All solvents and chemicals regents in the present work were of analytical grade and used without further purification.

### Method

First, 12 mg of MoO_3_ was dissolved in 10 ml of deionized water and stirred intensively for 10 min. Then 14 mg of thioacetamide, 0.12 g of urea and 0.68 g of NaCl were added to the above solution successively and stirred vigorously for another 2 h to form a homogeneous solution. Then the solution was transferred to a 50 ml Teflon lined stainless steel autoclave and loaded into an electric oven at 200 °C for 12 h. Finally, the black precipitate was collected by centrifuging, thoroughly washing with deionized water several times and freeze-drying. A part of the hydrothermal product was annealed in a tube furnace at 800 °C for 4 h in a nitrogen atmosphere for comparison.

### Characterization

The crystallography was investigated by X-ray diffractometer (XRD, Rigaku D/max 2500 pc, Cu Kα radiation: λ = 1.5406 Å). The morphology was characterized by field emission scanning electron microscopy (FESEM, JEOL JSM-6700F) and transmission electron microscopy (TEM, JEOL 2100F, 200 KV). The Micromeritics ASAP 2020 analyzer was used to determine the Brunauer-Emmett-Teller (BET) surface area and Barrett-Joyner-Halenda (BJH) porosities. The Raman spectra were obtained on WITec CRM200 confocal Raman microscopy system (WITec, Germany) with a laser wavelength of 488 nm. X-ray photoelectron spectroscopy (XPS) spectra were obtained on an ESCALAB Mk II (Vacuum Generators) spectrometer with monochromatized Al Kα X-rays (240 W).

### Electrochemical measurements

All the electrochemical measurements were performed with an electrochemical workstation (Princeton Applied Research). The working electrode was prepared by mixing the prepared active material, acetylene black and polyvinylidene fluoride (PVDF) binder in a weight ratio of 80:15:5 with N-methyl-2-pyrrolidinone (NMP) as a solvent. The resulting slurry was cautiously pasted onto a clean nickel foam (1 cm × 1 cm) substrate with a mass loading of 2.0 mg cm^−2^, and dried at 80 °C for 12 h in a vacuum. For three-electrode measurements, a platinum plate (Pt) was used as the counter electrode, and a saturated calomel electrode (SCE) was used as the reference electrode. Four electrolytes including 3 M KOH, 3 M NaOH, 3 M KCl, 0.5 M K_2_SO_4_ were used for comparison. Electrochemical impedance spectroscopy (EIS) measurements were performed in a frequency range from 0.01 to 100 kHz with 5 mV amplitude.

The hybrid supercapacitor was assembled into a device using the as-synthesized MoS_2_ electrode as the positive electrode, a commercial activated carbon (AC) electrode as the negative electrode and one piece of cellulose paper as a separator in 3 M KOH electrolyte. The preparation of the negative electrode was similar to that of the positive electrode. The mass ratio of MoS_2_ on the positive electrode to AC on the negative electrode was decided according to the well-known charge balance theory (q^+^ = q^−^). The charge stored (q) by each electrode depends on the following equation^9^:

2 $${\rm{q}}={\rm{m}}\times {\rm{C}}\times {\rm{\Delta }}{\rm{V}}$$

where C (F g^−1^) is the specific capacitance of the electrode, ∆V (V) is the potential window and m (g) is the mass loading. In order to get q^+^ = q^−^, the mass balancing will follow the equation^9^.

3 $${m}^{+}/{m}^{-}=({{\rm{C}}}^{-}\times {{\rm{\Delta }}{\rm{V}}}^{-})/({{\rm{C}}}^{+}\times {{\rm{\Delta }}{\rm{V}}}^{+})$$

Based on analysis of the potential windows and the specific capacitances of the MoS_2_ and AC electrodes from cyclic voltammograms (CVs) in Fig. S1 and galvanostatic charge-discharge curves (GCD) in Fig. S2, the optimal mass ratio of the positive electrode to the negative electrode was about 0.24 (the mass loading of the positive electrode is 2.0 mg cm^−2^ and that of the negative electrode is 8.3 mg cm^−2^). The electrochemical performance of the AC electrode in a three-electrode system in 3 M KOH is shown in Fig. S3. The calculation methodology on special capacitance, coulombic efficiency, energy density and power density were also shown in the supporting information.

## Supplementary information

Supporting Information
