# Supplementary material for: Charge Storage by Electrochemical Reaction of Water Bilayers Absorbed on MoS2 Monolayers
Source: Sci Rep. 2019 Mar 8;9:3980. doi: 10.1038/s41598-019-40672-w (PMC6408587; doi:10.1038/s41598-019-40672-w)
Supplement: Supplementary file 1 — Supporting Information [file 41598_2019_40672_MOESM1_ESM.docx]

Supporting Information

**Charge Storage by Electrochemical Reaction of Water Bilayers Absorbed on MoS_2_ Monolayers**

Ruihua Zhou^1, +^, Sufeng Wei^2, +^ , Yan Liu^3^, Nan Gao^4^, Guoyong Wang^1,*^, Jianshe Lian^1^and Qing Jiang^1^

^1^Key Laboratory of Automobile Materials, Department of Materials Science and Engineering, Jilin University, Changchun, 130025, PR China

^2^Key Laboratory of Advanced Structural Materials, Changchun University of Technology, Changchun, 130012, PR China

^3^Key Laboratory of Bionic Engineering (Ministry of Education) and State Key Laboratory of Automotive Simulation and Control, Jilin University, Changchun 130022, PR China

^4^College of Mechanical and Vehicular Engineering, Changchun University

Changchun, CN

*Correspondence: [materwanggy@jlu.edu.cn](mailto:materwanggy@jlu.edu.cn)

^+^These authors contributed equally to this work

**Calculation**

The special capacitance of the electrode can be calculated based on the CV and GCD

curves according to the following formulas:

$Cs=\frac{\int\mathrm{IdV}}{vm\Delta V}$ （1）

$C_{s}=\frac{I\times\triangle t}{m\times\triangle V}$ （2）

Where C_s_ (F g^-1^) is the special capacitance; m (g) is the mass of active materials on electrodes; $\triangle$V (V) is the is the effective potential range during discharging process;$v$ (V s^-1^) is the scan rate; I (A) is the discharge current;$\triangle$t (s) is the discharge time.

The coulombic efficiency of the systems was obtained from the following equation:

$\eta\left( \% \right)=\frac{{\Delta t}_{d}}{{\Delta t}_{c}}\times100$ (3)

Where ${\Delta t}_{d} (s)$is the discharge time and${\Delta t}_{c} (s)$ is the charge time.

The energy density and power density of the supercapacitors were calculated according to the following equations:

$E\left( Wh/kg \right)=\frac{0.5C_{T}{\Delta V}^{2}}{3.6}$ (4)

$P\left( W/kg \right)=\frac{3600E}{{\Delta t}_{d}}$ (5)

Where C_T_ is the total capacitance of the two-electrode cell; $\Delta V$ is the potential range during discharging process; ${\Delta t}_{d} (s)$is the discharging time; E is the energy density and P is the power density.


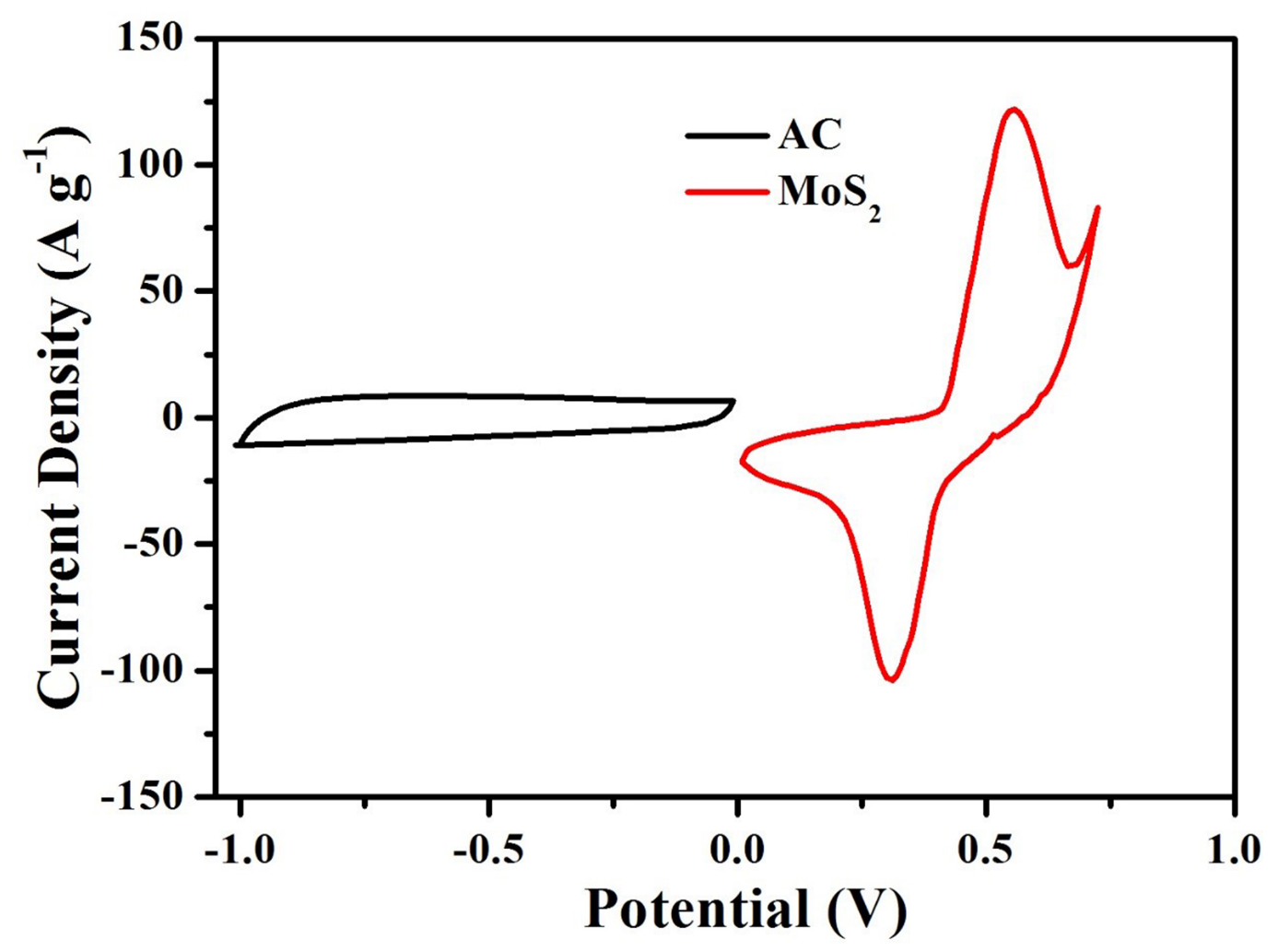


**Figure S1.** Comparative CV curves of AC and as-synthesized MoS_2_ nanosheets electrodes performed in a three-electrode configuration at a scan rate of 100 mV s^-1^ in KOH electrolyte.


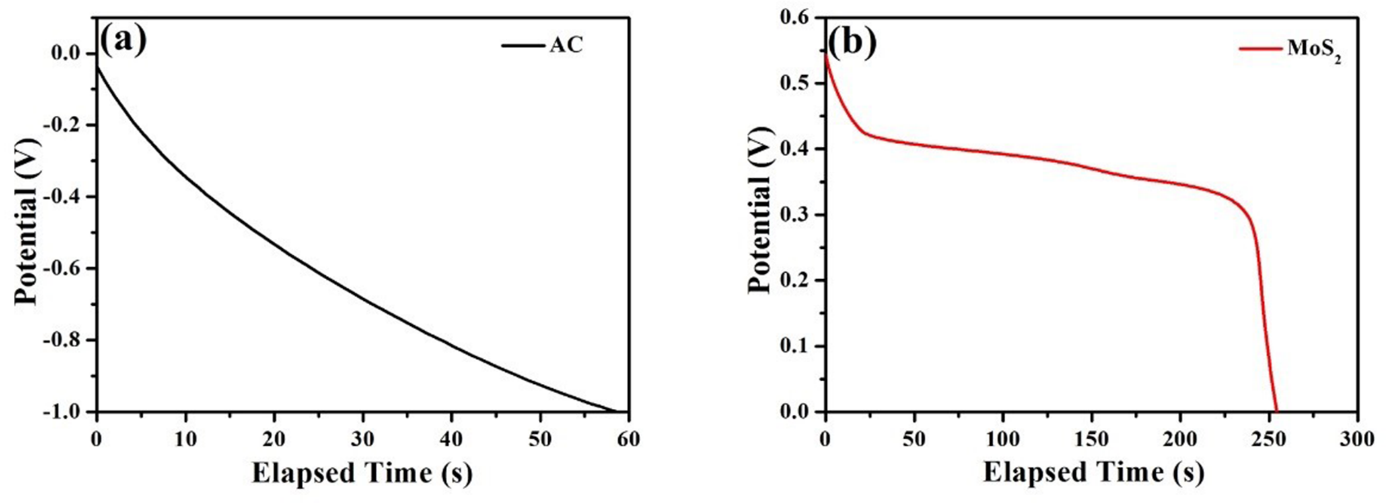


**Figure S2.** GCD curves of AC electrode (a) and as-synthesized MoS_2_ nanosheets electrode

(b) at a current density of 2 A g^-1^ in a three-electrode configurations in 3 M KOH electrolyte.


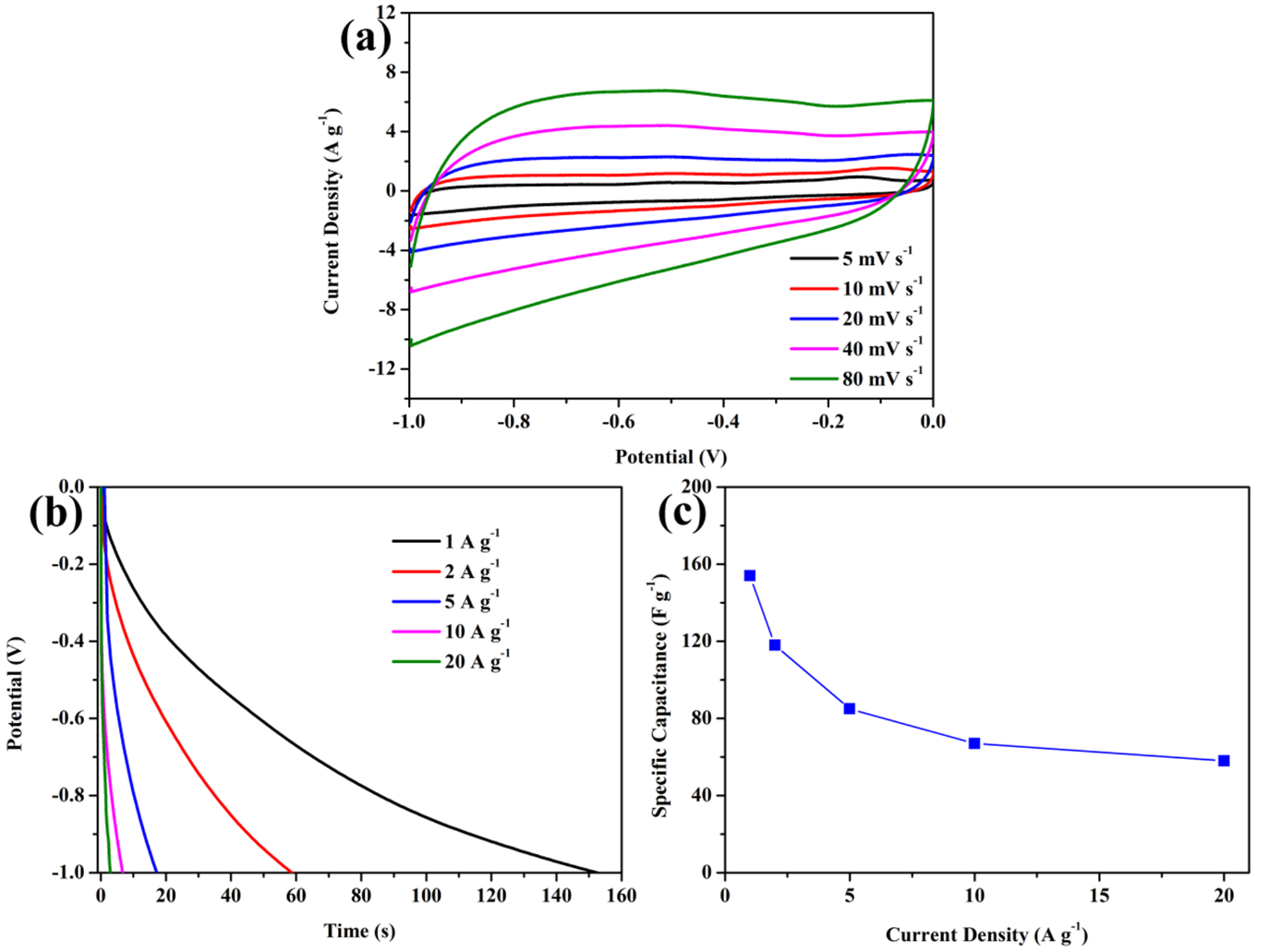


**Figure S3.** (a) CV curves; b) GCD curves of the AC electrode at different current densities in 3 M KOH, and (c) specific capacitance of AC as a function of current densities.

As shown in CV curves (Figure S3a), AC electrode exhibits the typical rectangular shapes from -1 to 0 V without a significant distortion even at a high scan rate of 80 mV s^-1^. According to the discharge curves (Figure S3b) using the formula (2), the AC electrode shows the specific capacitance of 154, 118, 85, 67 and 58 F g^-1^ at current densities of 1, 2, 5, 10 and 20 A g^-1^, respectively (Figure S3c).


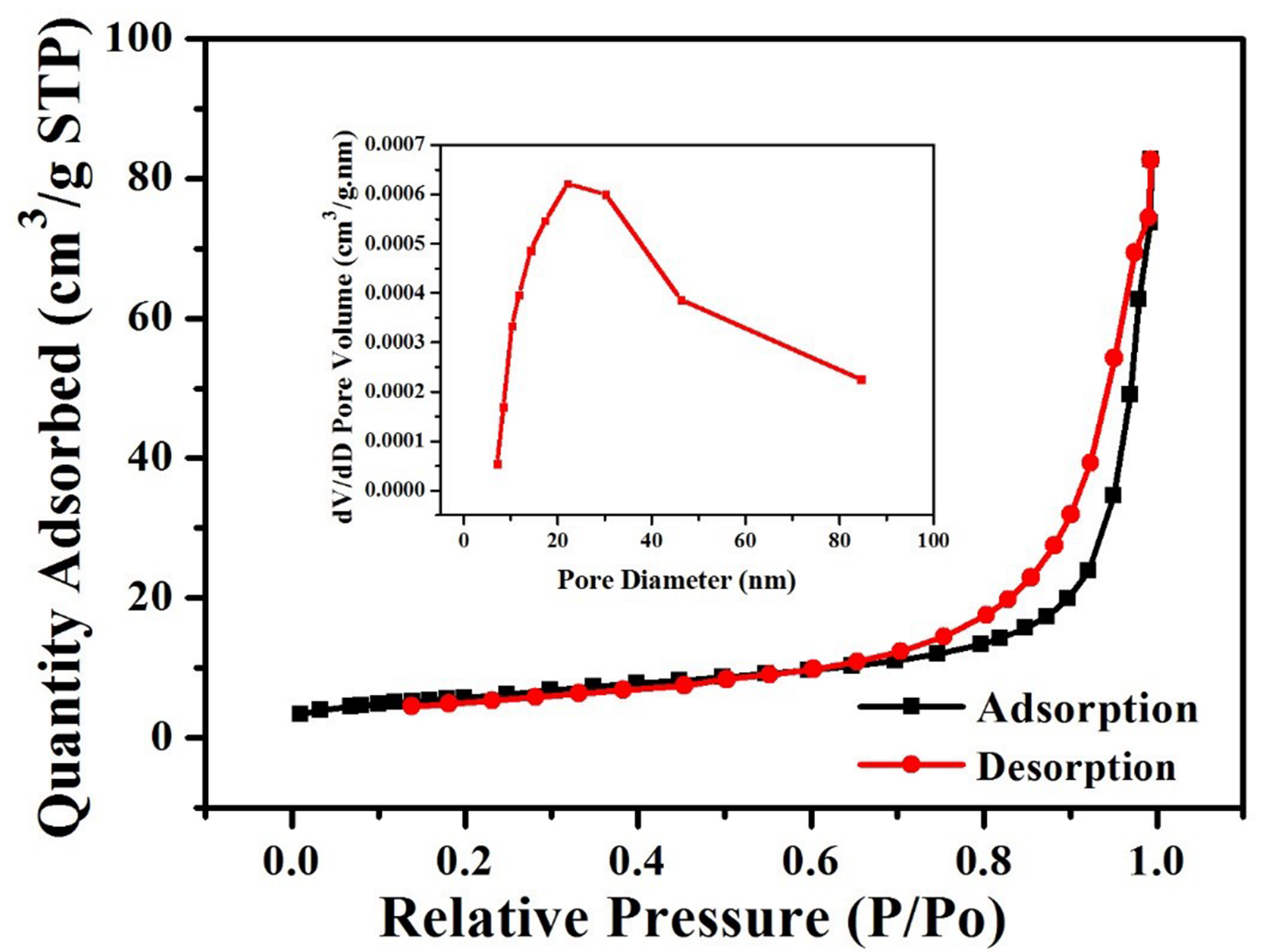


**Figure S4.** N_2_ adsorption-desorption isotherms and corresponding BJH pore-size distribution curves in the inset of the as-synthesized MoS_2_ nanosheets**.**


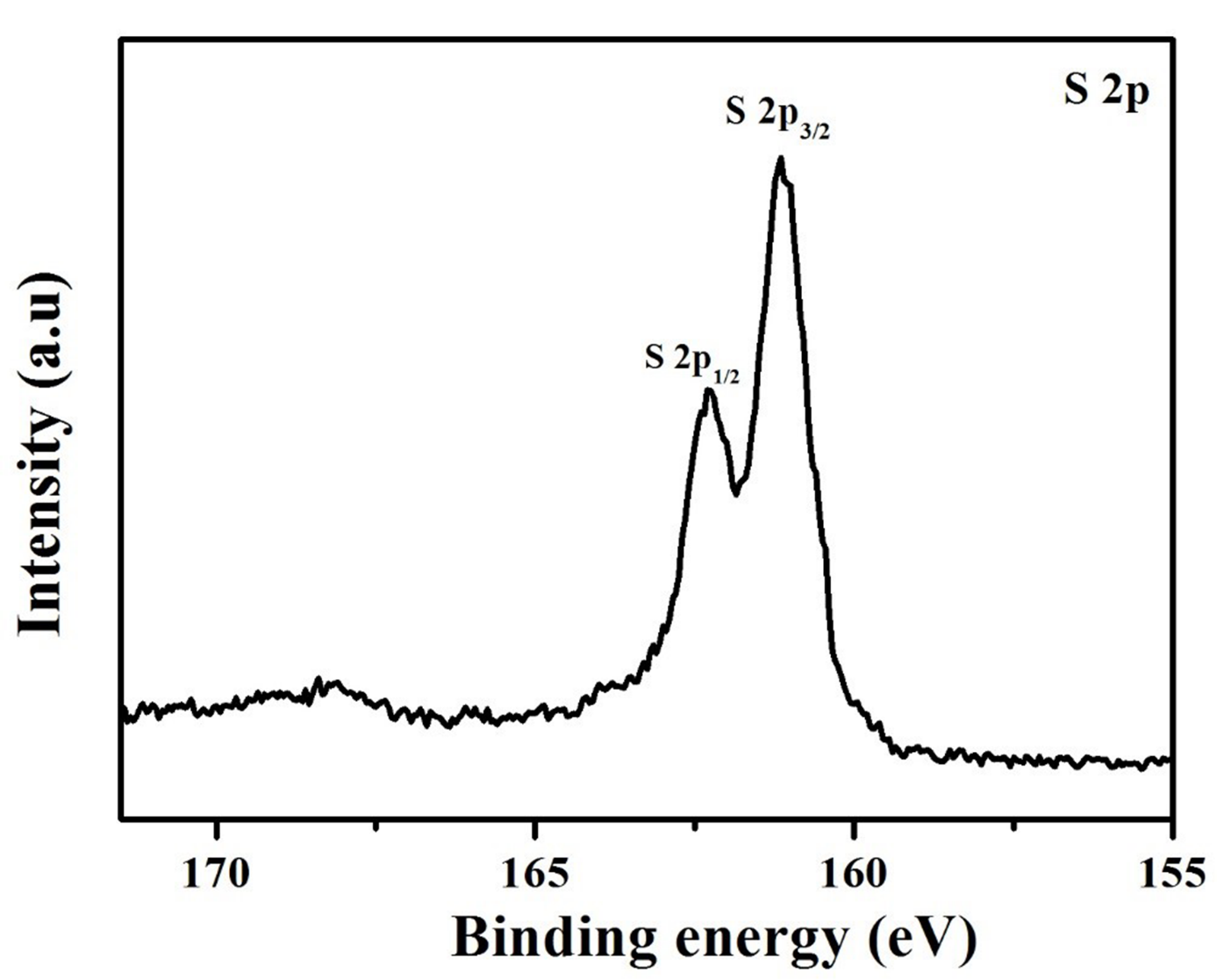


**Figure S5.** High-resolution X-ray photoelectron spectrum from S 2p region of the as-synthesized MoS_2_ nanosheets.


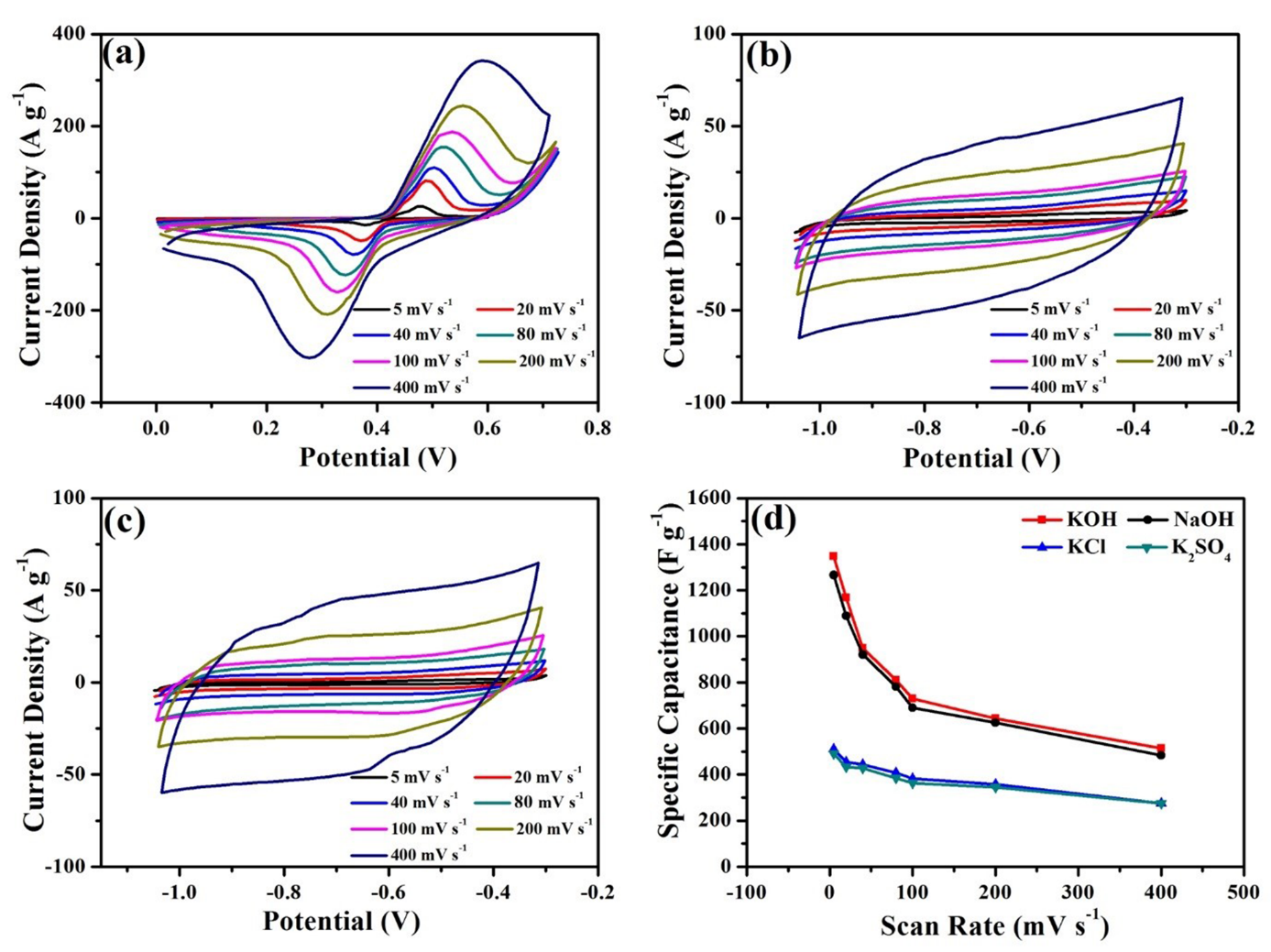


**Figure S6.** (a)-(c) CV curves of the as-synthesized MoS_2_ nanosheets at different scan rates in different electrolytes: (a) 3 M NaOH; (b) 3 M KCl; (c) 0.5 M K_2_SO_4_; (d) Specific capacitance at different scan rates in KOH, NaOH, KCl and K_2_SO_4_ electrolytes, respectively. The Cs of MoS_2_ in KOH is 1346 and 514 F g^-1^ at the scan rates of 5 and 400 mV s^-1^, respectively. The Cs of MoS_2_ in KCl is 510 and 274 F g^-1^ at the scan rates of 5 and 400 mV s^-1^, respectively.


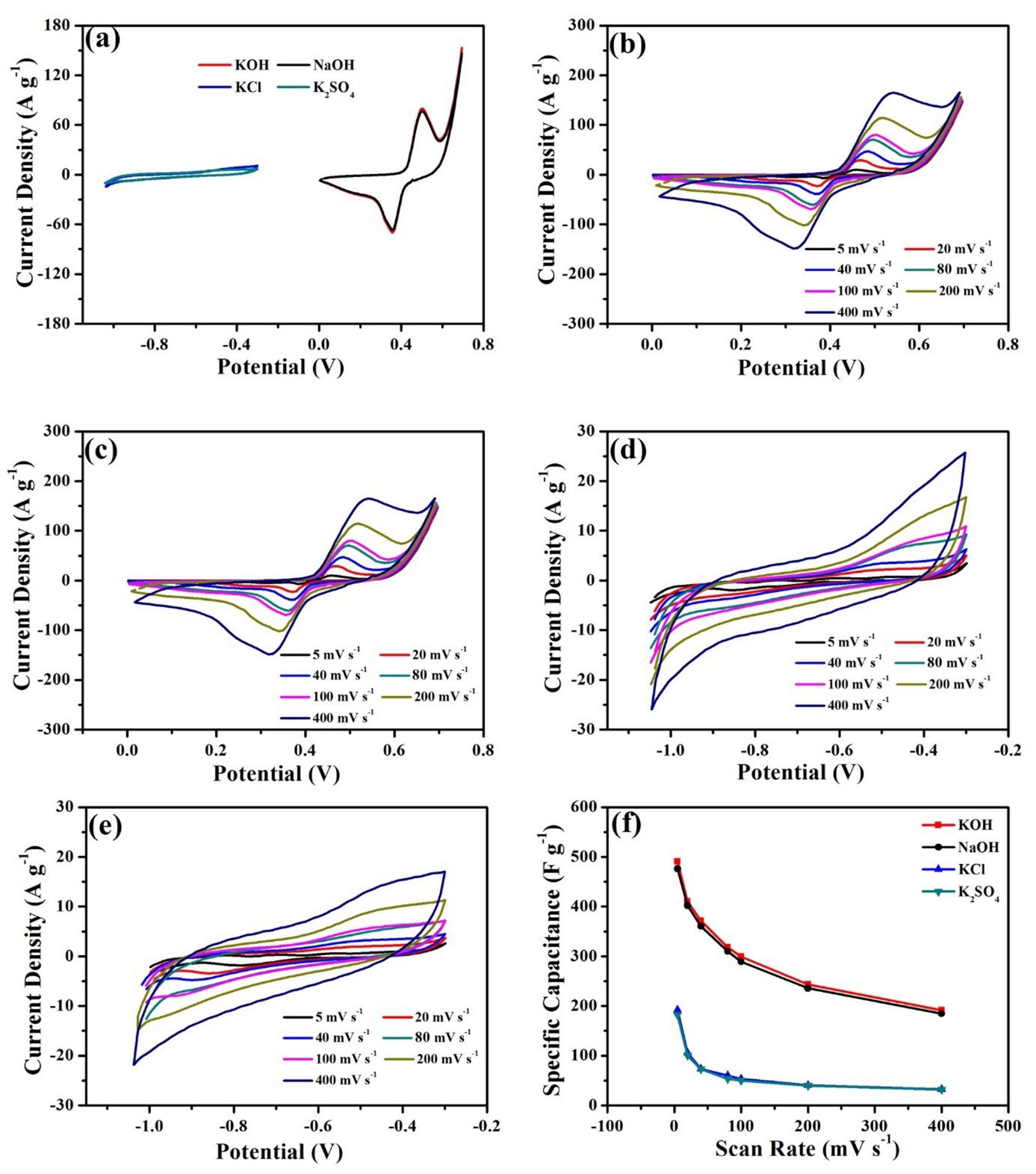


**Figure S7.** (a) Comparison of CV curves of annealed MoS_2_ nanosheets at a scanning rate of 100 mV s^-1^ in 3 M KOH, 3 M NaOH, 3 M KCl and 0.5 M K_2_SO_4_, respectively; (b)-(e) CV curves of annealed MoS_2_ nanosheets at different scan rates in different electrolytes: (b) KOH; (c) NaOH; (d) KCl; (e) K_2_SO_4_; (f) Specific capacitance at different scan rates in KOH, NaOH, KCl and K_2_SO_4_ electrolytes, respectively. The Cs of annealed MoS_2_ in KOH is 490 and 191 F g^-1^ at the scan rates of 5 and 400 mV s^-1^, respectively. The Cs of annealed MoS_2_ in KCl is 195 and 33 F g^-1^ at the scan rates of 5 and 400 mV s^-1^, respectively.


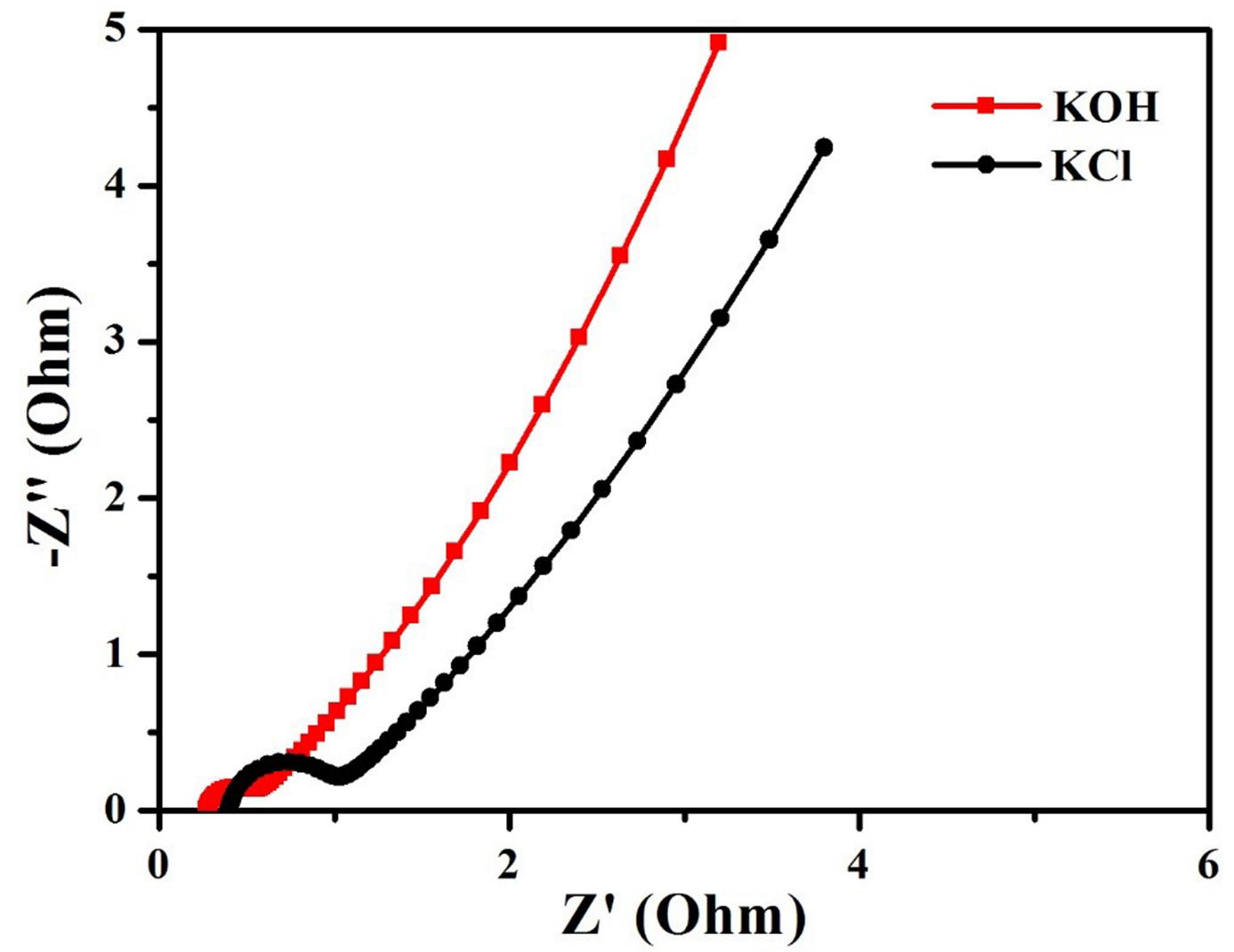


**Figure S8.** EIS of the as-synthesized MoS_2_ nanosheets in 3 M KOH and 3 M KCl electrolytes over the frequency range from 10^6^ to 10^-2^ Hz at room temperature, respectively.

**Table S1.** Energy storage performance comparison between our work and the previous reports.

| **Capacitor** | **Voltage** | **Specific Capacitance** | **Energy Density and Power Density** | **Reference** |
| --- | --- | --- | --- | --- |
| MoS_2_//AC | 1.7 V | 78.82 F g^-1^ at 0.5 A g^-1^ | 31.64 Wh kg^-1^ at 425 W kg^-1^ | our work |
| NiCo_2_O_4_@CNT/CNT | 2 V | 873 mF cm^-3^ at 0.5 A g^-1^ | 27.6 Wh kg^-1^ at 550 W kg^-1^ | Ref. 56 |
| CC-NC-LDH//AC | 1.6 V | 196 F g^-1^ at 1 A g^-1^ | 69.7 Wh kg^-1^ at 800 W kg^-1^ | Ref. 57 |
| NiMoO_4_//FeOOH | 1.7 V | 273 F g^-1^ at 1.5 A g^-1^ | 104.3 Wh kg^-1^ at 1270 W kg^-1^ | Ref. 58 |
| Ni_3_Se_2_@CF//AC | 1.5 V | 48.4 mAh g^-1^ at 1 A g^-1^ | 32.8 Wh kg^-1^ at 677.03 W kg^-1^ | Ref. 59 |
| Ni_3_S_2_/CoNi_2_S_4_/NF  //AC/NF | 1.6 V | 175 F g^-1^ at 1 A g^-1^ | 40 Wh kg^-1^ at 1730 W kg^-1^ | Ref. 60 |
| CC@NiCo_2_O_4_//CC@C | ------- | 89.7 F g^-1^ at 5 mA cm^-2^ | 31.9 Wh kg^-1^ at 2900 W kg^-1^ | Ref. 61 |
| FeCo_2_S_4_-NiCo_2_S_4_ | ------- | --------- | 46 Wh kg^-1^ at 1070.2 W kg^-1^ | Ref. 62 |
| NiCo-LDH/CFC | 1.7 V | 147.6 F g^-1^ at 1 A g^-1^ | 59.2 Wh kg^-1^ at 850 W kg^-1^ | Ref. 63 |
| MnCo_2_O_4_@Ni(OH)_2_//AC | 1.6 V | 141 F g^-1^ at 0.5 A g^-1^ | 48 Wh kg^-1^ at 1400 W kg^-1^ | Ref. 64 |
| CQDs/NiCo_2_O_4_//AC | 1.5 V | 88.9 F g^-1^ at 0.5 A g^-1^ | 27.8 Wh kg^-1^ at 128 W kg^-1^ | Ref. 65 |
| Ni(OH)_2_@3D Ni //AC | 1.3 V | 92.8 F g^-1^ at 1 A g^-1^ | 21.8 Wh kg^-1^ at 660 W kg^-1^ | Ref. 66 |
| C/CoNi_3_O_4_//AC | 1.8 V | 64.7 F g^-1^ at 1 mA cm^-2^ | 29.1 Wh kg^-1^ at 130.4 W kg^-1^ | Ref. 67 |
| Ni*_x_*Co_1-_*_x_* LDH–ZTO//AC | 1.2 V | 125.2 F g^-1^ at 0.88 A g^-1^ | 23.7 Wh kg^-1^ at 284.2 W kg^-1^ | Ref. 68 |
| Ni(OH)_2_/CNT/NF//AC | 1.8 V | 112.5 F g^-1^ at 2.5 mA cm^-2^ | 50.6 Wh kg^-1^ at 95 W kg^-1^ | Ref. 8 |
| NiCo_2_O_4_–RGO//AC | 1.4 V | 99.4 F g^-1^ at 0.5 A g^-1^ | 23.32 Wh kg^-1^ at 324.9 W kg^-1^ | Ref. 69 |
| H-CoO*_x_*@Ni(OH)_2_//RGO@Fe_3_O_4_ | 1.4 V | 112 F g^-1^ at 1.2 A g^-1^ | 45.3 Wh kg^-1^ at 1010 W kg^-1^ | Ref. 70 |
| NiCo_2_S_4_//C | 1.6 V | 341 mF cm^-2^ at 1 mA cm^-2^ | 22.8 Wh kg^-1^ at 160 W kg^-1^ | Ref. 71 |
| Ni_3_S_2_//AC | 1.8 V | 154.4 F g^-1^ at 2 A g^-1^ | 60.3 Wh kg^-1^ at 3600 W kg^-1^ | Ref. 72 |
| CoNi_2_S_4_//AC | 1.8 V | --------- | 33.9 Wh kg^-1^ at 409 W kg^-1^ | Ref. 73 |
| NiCo_2_O_4_@Co_0.33_Ni_0.67_(OH)_2_//CMK-3 | 1.6 V | 87.9 F g^-1^ at 5 mA cm^-2^ | 31.2 Wh kg^-1^ at 396 W kg^-1^ | Ref. 74 |
| Ni–Zn–Co oxide/hydroxide | 1.5 V | 69 F g^-1^ at 0.25 A g^-1^ | 16.62 Wh kg^-1^ at 2900 W kg^-1^ | Ref. 75 |
